# An Image-Free Single-Pixel Detection System for Adaptive Multi-Target Tracking

**DOI:** 10.3390/s25133879

**Published:** 2025-06-21

**Authors:** Yicheng Peng, Jianing Yang, Yuhao Feng, Shijie Yu, Fei Xing, Ting Sun

**Affiliations:** 1 School of Instrument Science and Opto-Electronic Engineering, Beijing Information Science and Technology University, Beijing 100192, China; pengyicheng@bistu.edu.cn (Y.P.); feng_yuhao22@bistu.edu.cn (Y.F.); yushijie@bistu.edu.cn (S.Y.); 2 Department of Precision Instrument, Tsinghua University, Beijing 100084, China; yangjn21@mails.tsinghua.edu.cn (J.Y.); xingfei@mail.tsinghua.edu.cn (F.X.)

**Keywords:** multi-target tracking, image-free, single-pixel detection

## Abstract

Conventional vision-based sensors face limitations such as low update rates, restricted applicability, and insufficient robustness in dynamic environments with complex object motions. Single-pixel tracking systems offer high efficiency and minimal data redundancy by directly acquiring target positions without full-image reconstruction. This paper proposes a single-pixel detection system for adaptive multi-target tracking based on the geometric moment and the exponentially weighted moving average (EWMA). The proposed system leverages geometric moments for high-speed target localization, requiring merely 3N measurements to resolve centroids for N targets. Furthermore, the output values of the system are used to continuously update the weight parameters, enabling adaptation to varying motion patterns and ensuring consistent tracking stability. Experimental validation using a digital micromirror device (DMD) operating at 17.857 kHz demonstrates a theoretical tracking update rate of 1984 Hz for three objects. Quantitative evaluations under 1920 × 1080 pixel resolution reveal a normalized root mean square error (NRMSE) of 0.00785, confirming the method’s capability for robust multi-target tracking in practical applications.

## 1. Introduction

Multi-target tracking technology is a core requirement in many modern applications, such as drone navigation, autonomous driving and remote sensing [1,2,3,4,5,6,7,8]. These tasks typically demand high localization accuracy, low latency, and energy-efficient hardware. Existing multi-target tracking systems are generally categorized into two groups: image-based and image-free. With the advancement of computer vision, image-based target tracking has become widely adopted in daily life due to its cost-effectiveness. High-speed cameras [9,10], which rely on image-based methods, are widely used to continuously capture scene images and extract targets for position estimation.

However, achieving high-precision localization with high-speed cameras often requires considerable computational resources. Higher precision requirements lead to more complex algorithms and increased processing time. In image-based tracking, the contradiction between temporal resolution and spatial resolution is a persistent challenge. Some researchers have focused on refining the hardware design, providing alternatives to traditional software processing, and working to mitigate the conflict between temporal and spatial resolution in target tracking [11,12,13]. Wei et al. [12] combined a line-by-line structure with an electronic rolling shutter technique, employing hardware logic circuits instead of software code for real-time information processing. Teman et al. [13] employed a CMOS sensor window readout mode to focus on the region of interest, thus reducing the computational burden. However, they were still limited to tracking targets through imaging, and the aforementioned conflict has not been effectively addressed. As a device capable of the high-frequency modulation of optical fields, the digital micromirror device (DMD) has attracted increasing attention [14,15,16]. Researchers have attempted to integrate it with a single-pixel detector for data acquisition, enabling image-free tracking [17,18,19]. Scholars have explored various coding masks to obtain the trajectory of the target without reconstructing the image [20,21,22]. Zhang et al. [21] used Fourier basis patterns to illuminate targets and captured the optical signals through a single-pixel detector, achieving an tracking update rate of up to 1666 Hz. Yang et al. [22] proposed a tracking method based on discrete cosine transform, achieving target tracking at only 0.59% of the Nyquist–Shannon sampling rate, reducing measurement time in complex backgrounds. Furthermore, some scholars have used geometric moments for centroid localization, significantly reducing the number of masks and improving the tracking update rate [23,24,25,26].

The rapid advancements in high-speed, high-precision single-target tracking with single-pixel detection have laid a solid foundation for further research in multi-target tracking. Some scholars extended the Fourier spectral properties to multi-target tracking [27,28,29]. Zhang et al. [28] established equations based on the correspondence between object displacement and Fourier phase, solving them to obtain multiple target positions for tracking. Yu et al. [29] optimized the selection of speckle patterns using Fourier spectrum characteristics, effectively reducing the sampling rate for localization and combining geometric moments for multi-target positioning. Some proposed projection methods to convert two-dimensional image information into projection curves, enabling multi-target localization [30,31]. Zheng et al. [31] designed two sets of patterns using a DMD, which were projected onto targets at different angles. The method calculated the positions of individual targets based on the obtained projection curves.

However, existing single-pixel-based multi-target localization systems face limitations such as low update rates, restricted applicability, and insufficient robustness in dynamic environments with complex object motions. In [31], in order to obtain the positions of multiple targets at a given moment, a large number of masks need to be projected. The number of masks required is related to the imaging size of the targets on the DMD. This process is time-consuming and limits the update rate of the method. In [28], the windowing method used suffers from some hysteresis and may not accurately track the target during rapid motion changes. The method maps target changes to the Fourier domain, treating horizontal and vertical displacements as unknowns in a system of nonlinear equations. However, Fourier-domain measurement errors are often unavoidable, and even small errors can cause significant deviations in the solution. These deviations may lead to results far from the actual values or, in some cases, no solution at all, reducing the method’s stability.

To address these challenges, we present an image-free multi-target tracking system based on single-pixel detection and an adaptive exponentially weighted moving average (EWMA) framework. The system employs DMD as a spatial light modulator to establish a direct mapping between modulation patterns and spatial moment values, thereby integrating sensing and computation into a unified process. This approach significantly reduces data storage requirements and computational latency compared to conventional image reconstruction methods. The tracking process begins by estimating the initial positions of multiple targets using Radon projections, which capture projection curves from multiple angular perspectives. The adaptive EWMA algorithm then generates dynamically updated tracking windows, which are applied in conjunction with a centroid-based moment localization method to extract the precise coordinates of each target. To further improve performance in scenarios involving closely spaced targets, we introduce an EWMA-based joint-window localization strategy. This method combines real-time measurements with motion predictions, continuously adjusting weighting parameters based on tracking discrepancies. The iterative update mechanism forms a positive feedback loop, enabling robust, high-precision tracking even under significant spatial interference. Experimental results demonstrate that the proposed system effectively performs the adaptive tracking of multiple targets across different rates and relative positions. For *N* targets, it requires only *3N* measurements to obtain the position of each target.

## 2. Principle and Method

### 2.1. Single-Pixel System Design for Multi-Target Tracking

**Figure 1:**
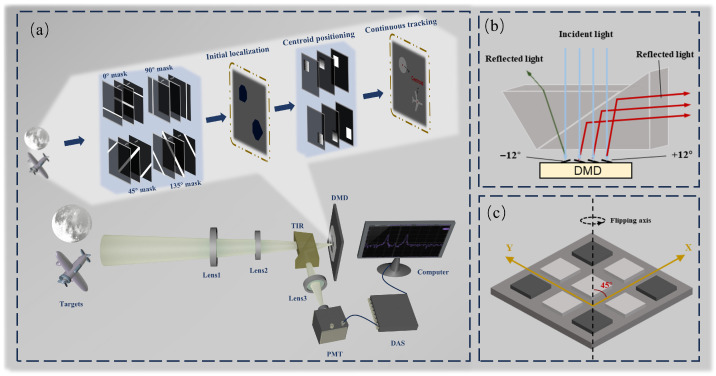
Scheme of the DMD-based mult-target tracking system. (**a**) System schematic. The core principle is to use a DMD to load encoding masks that modulate the light field of targets, while the crucial step involves performing angular projections and geometric moment-based centroid localization to achieve high-speed mult-target tracking via PMT signal acquisition. (**b**) Working principle of the TIR prism. (**c**) Schematic of micromirrors on DMD mounted at a 45° rotation angle.

The system schematic proposed in this paper is shown in Figure 1a. The core principle of the system is to use a DMD to load various encoded masks that modulate the optical field of the target. A photomultiplier tube (PMT) then collects the modulated light and converts it into an electrical signal, which is subsequently digitized and transferred to a computer for processing.

A crucial step in the measurement process is the generation of mask patterns by the DMD (Figure 1c), which modulates the light field of a scene containing multiple targets. Initially, the DMD performs projections of the targets from multiple angles, and the combined projection results are used to estimate the initial position of each target. Then, a window function is assigned to each target to separate them spatially. By integrating a geometric moment localization algorithm, the multi-target localization task is decomposed into several independent single-target localization problems. Due to the extremely high update rate of the photomultiplier tube (PMT), the time for each measurement is determined by the DMD’s flipping speed, reaching 56 µs.

However, the deflection angle generated by the DMD micromirrors is limited to only 24°, making it challenging to fully separate the incident and reflected light paths in the optical design. Extending the optical path to achieve separation would significantly increase the overall size of the system. To address this issue, this study introduces a total internal reflection (TIR) prism [32]. By efficiently redirecting the reflected light within the limited angular range, the TIR prism enables the effective spatial decoupling of the incident and reflected beams (Figure 1b). This not only solves the beam separation problem but also significantly improves the compactness and integration of the system. The proposed optical configuration provides strong support for the development of miniaturized, modular single-pixel detection systems.

### 2.2. DMD-Based Multi-Target Localization

#### 2.2.1. Initial Multi-Target Localization Based on Radon Projection

**Figure 2:**
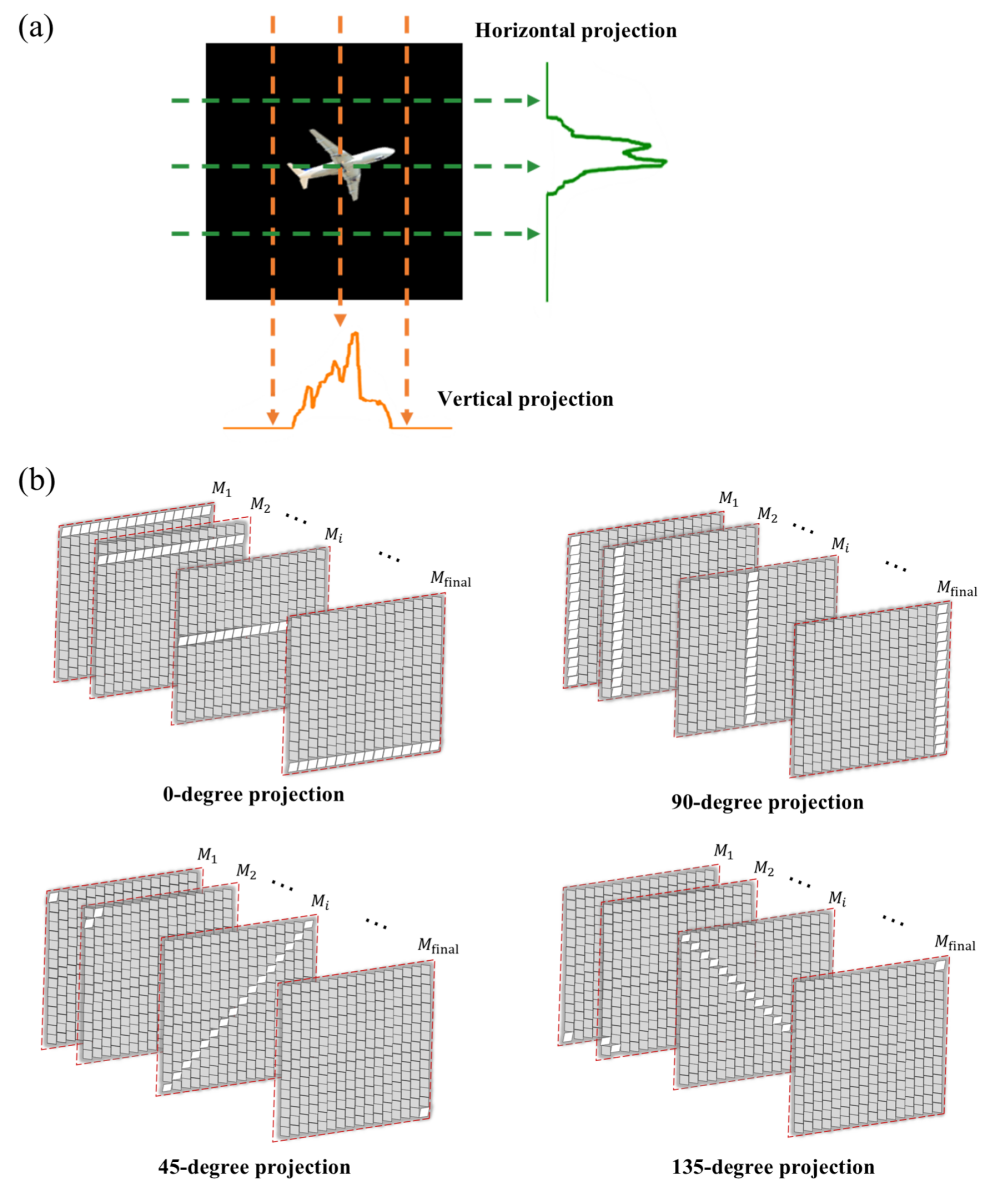
Radon projection based on DMD. (**a**) Schematic of the Radon projection principle. (**b**) Micromirror flip for 0-degree projection, 90-degree projection, 45-degree projection and 135-degree projection.

To obtain the initial positions of multiple targets, we use the Radon projection method for localization. Radon projection enables the transformation of high-dimensional image data into low-dimensional projection data. The core concept involves performing line integrals of a two-dimensional image along specific angles. This process sums the grayscale values of each pixel along the projection direction, generating one-dimensional projection values, as illustrated in Figure 2a.

The rapid flipping of micromirrors in the DMD enables projection modulation of the target light field from different angles. Micromirrors arranged at predefined angles flip in sequence, thereby scanning the entire DMD. As illustrated in Figure 2b, the DMD reflects the scene light field at 0°, 45°, 90°, and 135°, producing projection curves at these four angles. The scene light field containing the target is focused onto the DMD through the lenses. Micromirrors, arranged at predetermined angles, flip column by column to achieve modulation at the specified projection angle. The DMD modulates the light at this angle, which is collected by a photomultiplier tube and processed by a computer to generate the projection curve. This projection curve reflects the intensity variations at the corresponding positions in the image, providing information on the light intensity distribution.

By combining projection curves from multiple angles, the approximate positions of objects can be determined. We consider a specific region to contain an object if it shows values in projections from different angles. This approach is particularly useful for distinguishing between multiple objects, as the peaks in the projection curves help reveal the relative positions of the objects. For closely spaced targets, choosing projections from different angles helps mitigate the effects of proximity, thereby enhancing localization accuracy.

#### 2.2.2. Continuous Tracking via Geometric Moments

For a 2D continuous function *G(x,y)*, the formula for calculating its centroid in a Cartesian coordinate system can be expressed as follows:

(1) $$x_{c}=\frac{\;\int \int \;x\cdot G(x,y)dxdy}{\;\int \int \;G(x,y)dxdy}$$

(2) $$y_{c}=\frac{\;\int \int \;y\cdot G(x,y)dxdy}{\;\int \int \;G(x,y)dxdy}$$

where $x_{c}$ and $y_{c}$ represent the centroid coordinates of the desired region. The principle behind this calculation is to perform a weighted average over the spatial positions of the function. When the region of interest is a discrete area *D(x,y)*, the integral operations can be converted into summations. The centroid formulas are as follows:

(3) $$x_{c}=\frac{\sum_{x}\sum_{y}x\cdot D\left(x,y\right)}{\sum_{x}\sum_{y}D\left(x,y\right)}$$

(4) $$y_{c}=\frac{\sum_{x}\sum_{y}y\cdot D\left(x,y\right)}{\sum_{x}\sum_{y}D\left(x,y\right)}$$

When the target scene is imaged on the discrete micromirror array of the DMD, the scene’s light field *D(i,j)* represents the light intensity at position *(i,j)*. On a DMD of size *I × J*, *(i,j)* corresponds to the coordinates of each micromirror. By controlling the DMD to generate a position-encoded mask *L(i,j)*, each position’s light intensity *D(i,j)* serves as a weight. The modulation process is achieved by computing the weighted sum over the scene’s light field area. Position information in the x-axis and y-axis directions is represented by $L_{1}$ and $L_{2}$, respectively, as shown in the following equations:

(5) $$\begin{array}{c} L_{1}={\left[\begin{array}{cccc} 1 & 2 & \cdots & I\\ 1 & 2 & \cdots & I\\ \vdots & \vdots & \vdots & \vdots \\ 1 & 2 & \cdots & I \end{array}\right]}_{I\times J},L_{2}={\left[\begin{array}{cccc} 1 & 1 & \cdots & 1\\ 2 & 2 & \cdots & 2\\ \vdots & \vdots & \vdots & \vdots \\ J & J & \cdots & J \end{array}\right]}_{I\times J} \end{array}$$

**Figure 3:**
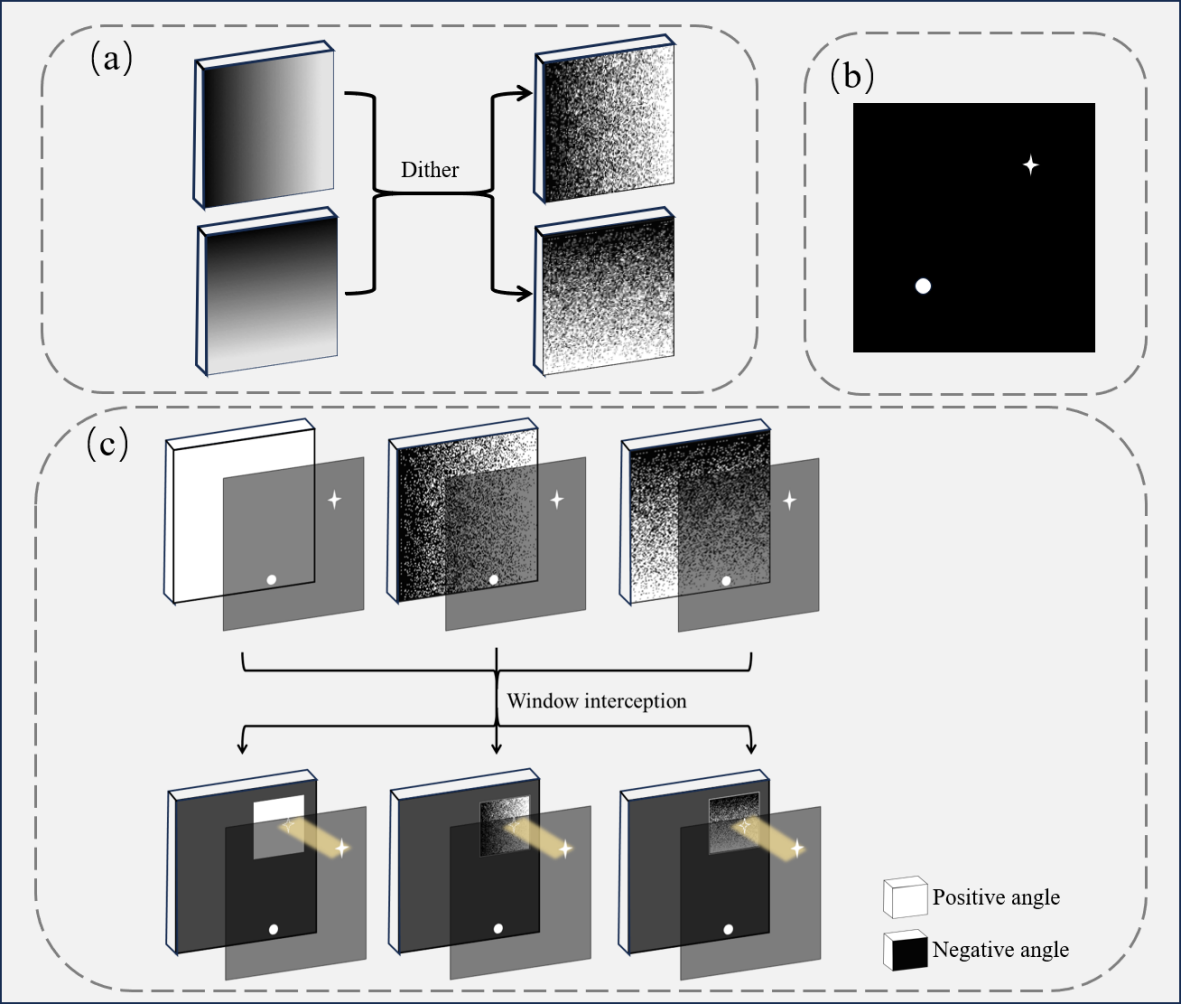
Window function for multi-target positioning. (**a**) Dithering for grayscale mask binarization. (**b**) Targets. (**c**) Window function to intercept multiple targets.

The above matrices can be represented by grayscale coding masks. However, the DMD can only generate a binary coding mask instead of a grayscale coding mask. To address this problem, we applied a spatial dithering method to produce grayscale coding masks [33]. This method quantizes the grayscale coding mask into 0 and 1 based on a threshold, and then the quantization error is diffused to adjacent pixels to reduce the error in local regions, achieving binarization of the grayscale mask, as shown in Figure 3a. The DMD is controlled to flip according to the binarized mask, with two flip angles corresponding to two gray values.

The centroid of the scene light field *D(i,j)* can be calculated as follows:

(6) $$x_{c}=\frac{\sum_{\omega }\left[L_{1}\left(i,j\right)\cdot D\left(i,j\right)\right]}{\sum_{\omega }D\left(i,j\right)}$$

(7) $$y_{c}=\frac{\sum_{\omega }\left[L_{2}\left(i,j\right)\cdot D\left(i,j\right)\right]}{\sum_{\omega }D\left(i,j\right)}$$

where ω represents the modulated window region on the DMD. By utilizing two encoded masks, $L_{1}$ and $L_{2}$, generated by the DMD, the scene light field *D(i,j)*, containing target information, is modulated. A photodetector sequentially collects and measures the light intensities, thereby achieving the summation operations in the formulas, with the modulated light intensities denoted as $S_{1}$ and $S_{2}$:

(8) $$S_{1}=\sum_{\omega }D\left(i,j\right)\cdot L_{1}\left(i,j\right),S_{2}=\sum_{\omega }D\left(i,j\right)\cdot L_{2}\left(i,j\right)$$

(9) $$S_{3}=\sum_{\omega }D\left(i,j\right)$$

After the DMD rapidly flips to modulate the light field, the photodetector instantly receives the reflected light and calculates the centroid of the target. The calculated centroid coordinates $\left(x_{c},y_{c}\right)$ can be expressed as follows:

(10) $$x_{c}=\frac{S_{1}}{S_{3}},\;y_{c}=\frac{S_{2}}{S_{3}}$$

### 2.3. Multi-Target Window Tracking Method Based on Adaptive EWMA

When the light field containing multiple targets is projected onto the DMD, the geometric moment calculation will yield the average centroid of multiple objects. To acquire the individual positions of each target, we propose a window-based geometric moment (WGM) localization method, converting the multi-target localization problem into multiple single-target localization problems. We first obtain each target’s initial position using the Radon projection method mentioned earlier and then apply a window around each target’s corresponding position. As shown in Figure 3c, white dots on the DMD represent micromirrors flipped to a positive angle, while black dots represent those flipped to a negative angle. The photodetector is positioned to receive only light reflected by micromirrors set to a positive angle. A rectangular window is centered on the target’s initial location, and a new modulation mask is generated within this window on the DMD. Micromirrors outside the window are flipped to a negative angle to isolate the interference of light intensity from other targets. The modulation can be expressed as

(11) $$S_{w1}=\sum_{\omega }D\left(i,j\right)\cdot w\left(i,j\right)\cdot L_{w1}\left(i,j\right)$$

(12) $$S_{w2}=\sum_{\omega }D\left(i,j\right)\cdot w\left(i,j\right)\cdot L_{w2}\left(i,j\right)$$

(13) $$S_{w3}=\sum_{\omega }D\left(i,j\right)\cdot w\left(i,j\right)$$

where *w(i,j)* is the window function and $L_{w_{1}}\left(i,j\right)$ and $L_{w_{2}}\left(i,j\right)$ are the grayscale masks generated in the window. Each target only requires three flips to determine its corresponding position. We employ the centroid position as the center of the window function at the next moment, which allows continuous tracking.

**Figure 4:**
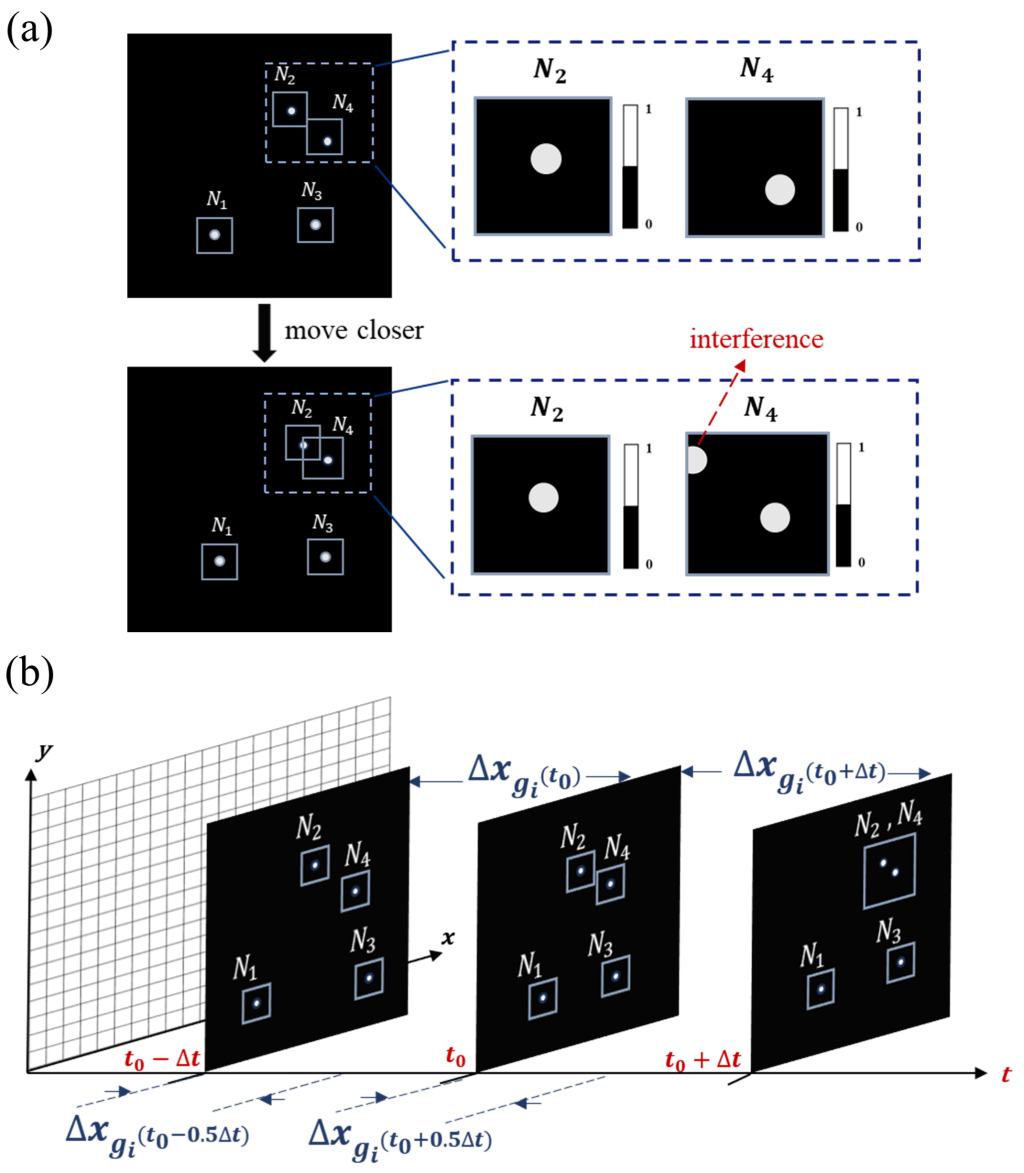
Multi-target tracking under close-range interference. (**a**) Interference from target proximity on window function positioning. (**b**) Schematic of close-range mult-target tracking.

However, this tracking method cannot track fast-moving targets due to its lag. Furthermore, when the targets come close to each other, multiple objects may appear within a single window, causing interference, as shown in Figure 4a. To address this problem, we proposed an adaptive multi-target window tracking method based on EWMA. EWMA is a statistical technique that smoothes measurements and predictions, often used to monitor changing processes [34]. Leveraging the fast flipping frequency of the DMD, it can be assumed that the speed ratio of the target over a short period of consecutive measurements remains constant. Therefore, we can make a prediction based on the previous speed and obtain a predicted position. When objects are far apart, we can allocate weights between the measured centroid $P_{obj}\left(t_{i}\right)$ and the predicted centroid $P_{obj}^{\prime }\left(t_{i+1}\right)$ according to the EWMA weighting parameter. The computed value is taken as $P_{window}\left(t_{i+1}\right)$, which serves as the center of the window function for the next moment, eliminating lag. In addition, the weight parameter is then adjusted based on the difference between the subsequent measured centroid $P_{obj}\left(t_{i+1}\right)$ and $P_{window}\left(t_{i+1}\right)$, achieving positive feedback. When the targets come close to each other, we propose a joint tracking method combined with EWMA, which effectively mitigates interference and allows for stable tracking. The derivation of joint tracking is as follows.

For a scene with *N* targets, at time $t_{0}$, we predict that *n* targets might move to a close proximity at $t_{0}+\Delta t$, causing them to appear in the same window. Consider Δt as a detection cycle and assume that it takes $T_{0}$ for the DMD to flip once. Then, a detection cycle can be expressed as

(14) $$\Delta t=N\cdot 3T_{0}$$

At the moment $t_{0}$ and before is the case where there is only one object in the window and the positions of all objects are known. And after the moment $t_{0}$, these *n* objects move to a window where the centroid of each object cannot be obtained directly. Assume that the *n* objects are $g_{1}$ to $g_{n}$, where $g_{i}$ denotes the i−th object. Taking the horizontal direction as an example, the average horizontal displacement of *n* objects in the time period $\left(t_{0},t_{0}+\frac{q_{j}}{N}\cdot \Delta t\right)$ is $\Delta \bar{x}\left(t_{0}+\frac{q_{j}}{N}\cdot \Delta t\right)$ denotes the serial number of the time period corresponding to each of these *n* measurements in *N*. Then similarly, $\Delta \bar{x}\left(t_{0}-\Delta t+\frac{q_{j}}{N}\cdot \Delta t\right)$ denotes the average horizontal displacement of *n* objects in the time period $\left(t_{0}-\Delta t,t_{0}-\Delta t+\frac{q_{j}}{N}\cdot \Delta t\right)$.

Because of the high flip-frequency of the DMD, two adjacent displacements at the same time interval Δt can be considered to be in a fixed proportion, as in the following equation:

(15) $$\begin{array}{cc} k_{g_{i}}\left(t_{0}+\Delta t\right) & =\frac{\Delta x_{g_{i}}\left(t_{0}+\frac{q_{j}}{N}\cdot \Delta t\right)}{\Delta x_{g_{i}}\left(t_{0}-\Delta t+\frac{q_{j}}{N}\cdot \Delta t\right)} \end{array}$$

For object $g_{i}$, its displacement during the measurement process is distributed proportionally across multiple intervals, represented by $q_{j}$, where $q_{j}$ takes values from 1 to *N*. The parameter *j* ranges from 1 to *n*, indicating the number of proportional displacement measurements. The value $q_{j}$ divides the time span into fractional steps within the measurement intervals. The displacement $\Delta x_{g_{i}}\left(t_{0}+\frac{q_{j}}{N}\cdot \Delta t\right)$ corresponds to the motion of $g_{i}$ during each of these *n* measurements relative to $t_{0}$, while $\Delta x_{g_{i}}\left(t_{0}-\Delta t+\frac{q_{j}}{N}\cdot \Delta t\right)$ represents the displacement during the previous interval Δt relative to $\left(t_{0}-\Delta t\right)$. These proportional displacements, spaced by Δt, form *n* pairs, and their ratio is denoted as $k_{g_{i}}\left(t_{0}+\Delta t\right)$. Since the displacement $\Delta x_{g_{i}}\left(t_{0}-\Delta t+\frac{q_{j}}{N}\cdot \Delta t\right)$ belongs to the known time before $t_{0}$, it can be multiplied by the ratio $k_{g_{i}}\left(t_{0}+\Delta t\right)$ to estimate the unknown displacement $\Delta x_{g_{i}}\left(t_{0}+\frac{q_{j}}{N}\cdot \Delta t\right)$.

And after the moment $t_{0}$, we can only obtain the mean horizontal centroid of the *n* objects at the moment $\left(t_{0}+\frac{q_{j}}{N}\cdot \Delta t\right)$ through the large window, and subtract it from the mean horizontal centroid at the moment $t_{0}$ to obtain the mean displacement $\Delta \bar{x}\left(t_{0}+\frac{q_{j}}{N}\cdot \Delta t\right)$ in this time period. The expression for the mean displacement is then obtained from the centroid formula:

(16) $$\begin{array}{cc} \; & \Delta \bar{x}\left(t_{0}+\frac{q_{j}}{N}\cdot \Delta t\right)=\\ \; & \frac{S_{g_{1}}\cdot \Delta x_{g_{1}}\left(t_{0}+\frac{q_{j}}{N}\cdot \Delta t\right)+\cdots +S_{g_{n}}\cdot \Delta x_{g_{n}}\left(t_{0}+\frac{q_{j}}{N}\cdot \Delta t\right)}{S_{g_{1}}+\cdots +S_{g_{n}}} \end{array}$$

where $S_{(}g_{i})$ is the reflected light intensity of the target $g_{i}$, and this is used as a weight to calculate the average displacement of all *n* objects in the time period $\left(t_{0},t_{0}+\frac{q_{j}}{N}\cdot \Delta t\right)$. The object $g_{i}$ corresponds to the parameter $k_{g_{i}}\left(t_{0}+\Delta t\right)$, and the associated (15) and (16) yield *n* equations containing *n* parameters *k*. The parameter $k_{g_{i}}\left(t_{0}+\Delta t\right)$ corresponding to the object $g_{i}$ is obtained by solving the system of *n* elemental equations, and the back-substitution into (15) yields the calculated values of the displacements:

(17) $$\Delta x_{g_{i}}{\left(t_{0}+\Delta t\right)}_{mea}=k_{g_{i}}\left(t_{0}+\Delta t\right)\cdot \Delta x_{g_{i}}\left(t_{0}-\Delta t\right)$$

The horizontal displacement of object $g_{i}$ in the time period $\left(t_{0},t_{0}+\Delta t\right)$ is finally obtained, thus achieving independent tracking in the case of multiple objects in one window. However, the method of solving the equation to obtain the target displacement requires high accuracy of the known parameters in the arithmetic equation. If the measured value obtained through the centroid moment has a large error with the real value, the solution of the equation is prone to error accumulation and becomes a wrong solution. In this regard, the experiment corrects the measured values according to the proposed adaptive EWMA method.

Experimentally, the trajectory of object $g_{i}$ before the moment $t_{0}$ is recorded, and a prediction value $\Delta x_{g_{i}}{\left(t_{0}+\Delta t\right)}_{pre}$ is obtained based on the trajectory. And the computed value $\Delta x_{g_{i}}\left(t_{0}+\Delta t\right)$ obtained by solving the equations will be weighted with the prediction value as per the following equation,

(18) $$\begin{array}{cc} \Delta x_{g_{i}}{\left(t_{0}+\Delta t\right)}_{real} & =\alpha_{g_{i}}\left(t_{0}+\Delta t\right)\cdot \Delta x_{g_{i}}{\left(t_{0}+\Delta t\right)}_{mea}+\\ \; & \left(1-\alpha_{g_{i}}\left(t_{0}+\Delta t\right)\right)\cdot \Delta x_{g_{i}}{\left(t_{0}+\Delta t\right)}_{pre} \end{array}$$

where $\alpha_{g_{i}}\left(t_{0}+\Delta t\right)$ is the smoothing coefficient for object $g_{i}$ during the time interval $\left(t_{0},t_{0}+\Delta t\right)$ and the value range is (0,1]. In practice, the smoothing coefficient is adaptively adjusted according to the motion dynamics of the target. For instance, when the object exhibits rapid or abrupt motion changes, increasing the value of the smoothing coefficient allows the system to respond more promptly to the measurement, thereby better adapting to fast variations. $\Delta x_{g_{i}}{\left(t_{0}+\Delta t\right)}_{real}$ is used as the final displacement value. In order to make the obtained value closer to the real value, the smoothing coefficient will be continuously adjusted according to the difference between the measured value and the predicted value as follows:

(19) $$\begin{array}{cc} \alpha_{g_{i}}\left(t_{0}+2\Delta t\right)= & \gamma \cdot \left|\Delta x_{g_{i}}{\left(t_{0}+\Delta t\right)}_{mea}-\Delta x_{g_{i}}{\left(t_{0}+\Delta t\right)}_{pre}\right|\\ \; & +\alpha_{g_{i}}\left(t_{0}+\Delta t\right) \end{array}$$

where $\alpha_{g_{i}}\left(t_{0}+2\Delta t\right)$ is the smoothing coefficient of object $g_{i}$ in the horizontal direction in the next time period and γ is the gain factor. If the difference between the calculated value and the predicted value is large, it means that the motion state of object $g_{i}$ in the horizontal direction changes drastically. Increasing the smoothing coefficient at the next moment allows the system to respond faster to changes in the measured value.

If the difference between the calculated value and the predicted value is small, it means that the change in the motion state of object $g_{i}$ in the horizontal direction is gentle and the system is updated smoothly. Varying the smoothing coefficient adaptively allows the values to be taken closer to the real situation. The same calculation procedure can be obtained for the vertical direction.

The four objects are tracked locally, as shown in Figure 4b. At $t_{0}$, we predict from the trajectory that window $N_{2}$ and window $N_{4}$ will cross in the next detection cycle. The system will not be able to obtain the centroid of the two objects in the next cycle directly. We turn the two small windows into a large one and calculate the average centroid of the two objects instead.

**Figure 5:**
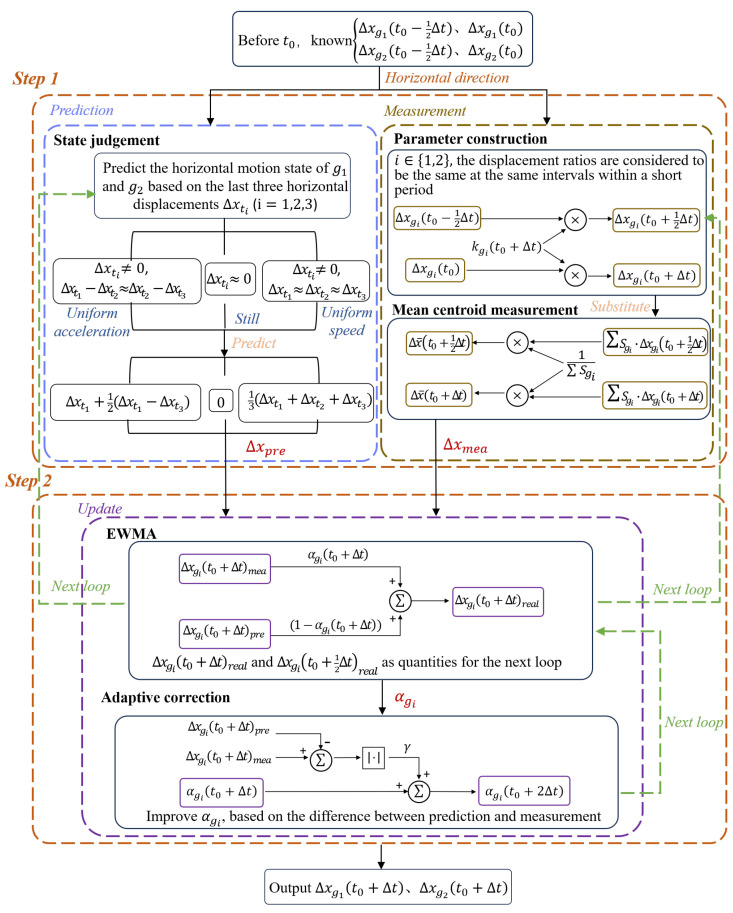
Multi-target tracking algorithm based on adaptive EWMA.

The objects in windows $N_{2}$ and $N_{4}$ are designated as $g_{1}$ and $g_{2}$, respectively. The flowchart for the adaptive EWMA method is shown in Figure 5, illustrating the tracking process of the horizontal positions of $g_{1}$ and $g_{2}$.

The first step involves measurement and prediction. Firstly, the scaling parameters are set based on the displacement values of the previous detection cycle. We use the displacement of a past time period to represent the displacement of a future time period. Since $N_{2}$ and $N_{4}$ are within the same window, centroid measurements of $g_{1}$ and $g_{2}$ are taken at the midpoint and end of the detection cycle, yielding the average centroids of $g_{1}$ and $g_{2}$ at these two moments. Then, the differences in average centroids relative to $t_{0}$ yield the average centroid displacements of the two objects during the intervals $\left(t_{0},t_{0}+\frac{1}{2}\Delta t\right)$ and $\left(t_{0},t_{0}+\Delta t\right)$. Two equations for the average centroid displacements are formulated, weighted by the total reflected light intensities of $g_{1}$ and $g_{2}$. The two unknowns are the scale parameters set beforehand. The system of equations is solved to find the displacement $\Delta x_{mea}$ of each individual object. Meanwhile, the motion state of the object is judged according to the prior three detection cycles, so as to predict the displacement $\Delta x_{pre}$. In the second step, the adaptive EWMA method is employed to obtain a displacement value that is closer to the true value. An appropriate smoothing factor is chosen from (18) to allocate weights to both the measured and predicted values. Then, the smoothing factor is improved according to the difference between the measured and predicted values for the next moment. Finally, the horizontal displacements of $g_{1}$ and $g_{2}$ over the respective time intervals are obtained, yielding outputs $\Delta x_{g1}\left(t_{0}+\Delta t\right)$ and $\Delta x_{g2}\left(t_{0}+\Delta t\right)$. Additionally, $\Delta x_{g1}\left(t_{0}+\frac{1}{2}\Delta t\right)$ and $\Delta x_{g2}\left(t_{0}+\frac{1}{2}\Delta t\right)$ are calculated and used for the next prediction and measurement cycle, enabling continuous high-precision tracking.

Therefore, the proposed method continuously monitors the relative distance between objects, enabling the adaptive tracking of multiple moving targets. For a DMD with a flip frequency of *f*, the update rate of localization for *N* targets can be achieved up to $\frac{f}{3N}$.

## 3. Simulation and Experiment

### 3.1. Simulation

#### 3.1.1. Radon Projection

**Figure 6:**
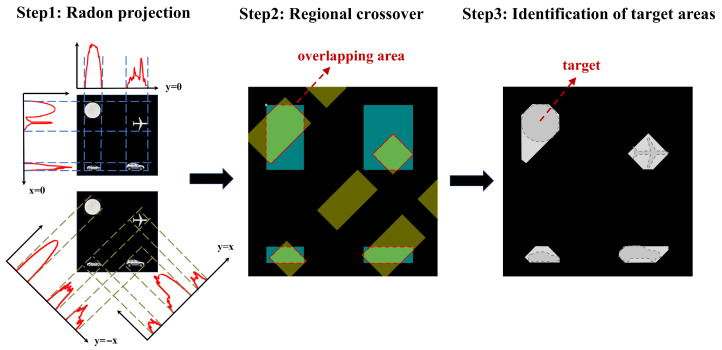
Radon projection for mult-target initial localization.

The process of Radon projection is shown in Figure 6. Firstly, Radon projection is performed on the target from angles of 0°, 45°, 90°, and 135°. The projection curves of the target region are obtained from multiple angles. Then, region extraction is performed on the basis of the intensity of these curves. Regions with non-zero projection values indicate the presence of a target at those angles. Subsequently, areas that overlap from different angles are marked. Positions where the projection curves exhibit non-zero values across all four angles are identified as target areas.

We measure the target dimensions on the basis of the size of the identified target region. The center position of the window function corresponding to the target is determined by averaging the projection region’s values in the horizontal and vertical directions. The measurement results are shown in Table 1. The results indicate that the center of the window is close to the object’s centroid. Selecting an appropriately sized window ensures that the target remains fully captured.

#### 3.1.2. Multi-Target Window Tracking Method Based on Adaptive EWMA

**Figure 7:**
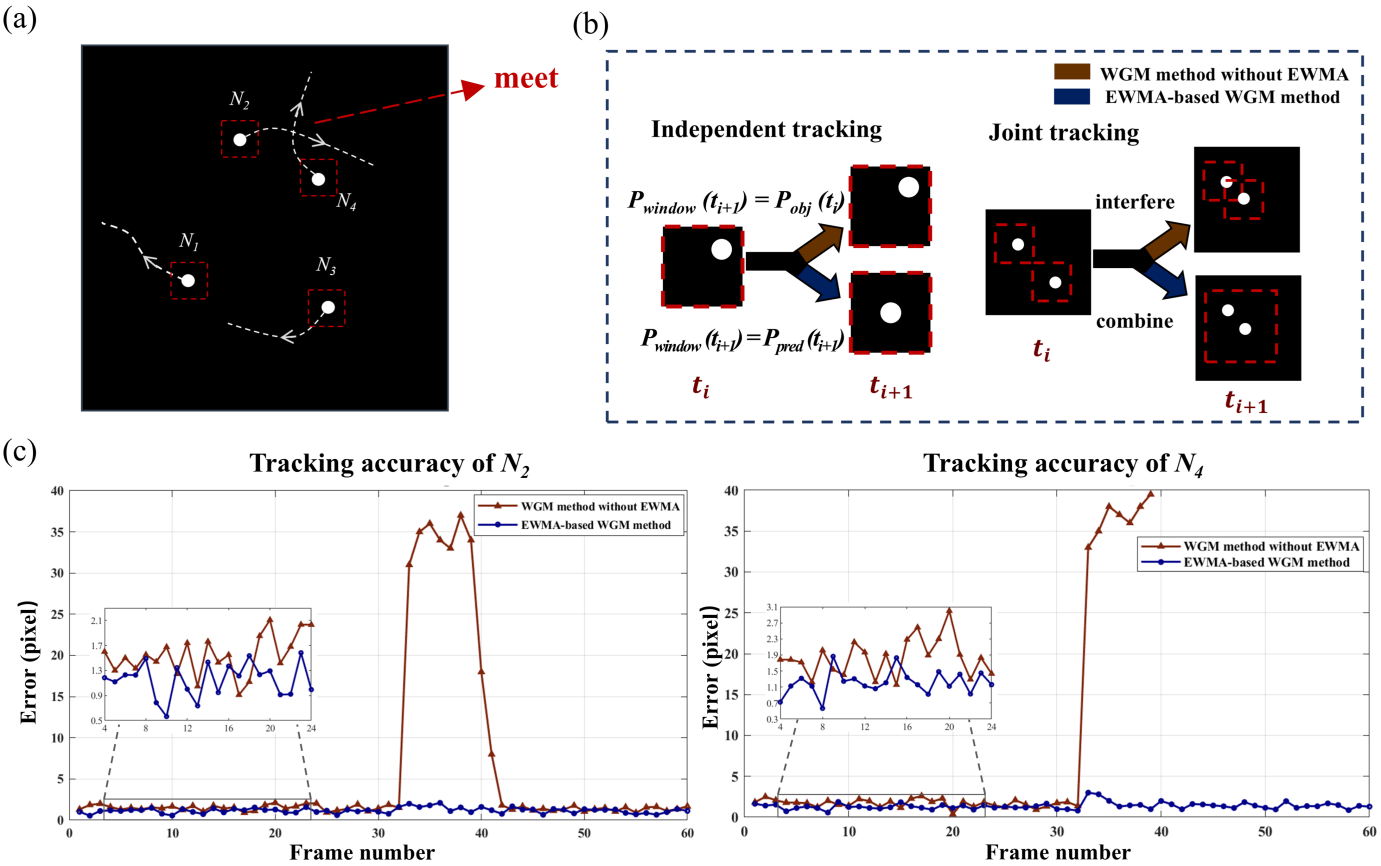
Simulation of mult-target tracking using the EWMA-based WGM method. (**a**) Target trajectories. (**b**) Tracking principle of EWMA-based WGM method in two different scenarios. (**c**) Tracking accuracy of targets $N_{2}$ and $N_{4}$ using WGM method with and without EWMA.

Upon ascertaining the positions of multiple targets, the subsequent tracking can be achieved using the WGM localization methodology. Considering the varying distances between targets, we categorize the tracking into independent tracking and joint tracking. When the targets are far apart, they do not interfere with each other, and each window function corresponds to a single target for independent tracking. When the targets are close together, multiple targets may appear within a single window, making it impossible to directly calculate the centroid of each target. In this case, we employed the EWMA-based WGM method to track continuously, which combined the information of targets for computation. In order to validate the proposed method, we performed a tracking simulation of multiple moving objects, which was realized by sequentially inputting 60 pictures. There are 4 objects in the picture, each with a diameter of 40 pixels, and the motion trajectories they exhibit are shown in Figure 7a. During the motion, objects $N_{2}$ and $N_{4}$ will be in close in proximity to each other.

We use the improved WGM-based localization method for tracking, which integrates the adaptive EWMA principle, as illustrated in Figure 7b. In independent tracking, the EWMA-based WGM method predicts the position at $t_{i+1}$ using the trajectory before $t_{i}$, ensuring that the target remains centered within the window rather than outside it. In joint tracking, the EWMA-based WGM method dynamically detects the distance between objects. When objects come close, two smaller windows merge into a larger one, integrating multiple data sources for computation and localization.

We track four objects, and the localization accuracy of objects $N_{2}$ and $N_{4}$ is illustrated in Figure 7c. The two objects meet at frame 33 and separate at frame 40. Prior to their encounter, both methods demonstrate stable and reliable tracking performance. As the objects approach each other, the standalone WGM method fails to maintain continuous tracking and eventually loses track of object $N_{4}$. In contrast, the EWMA-based WGM method ensures stable and high-precision tracking throughout the process. The tracking results for the four moving targets are shown in Table 2. It is evident that the EWMA-based WGM method exhibits high robustness regardless of the relative distances between the targets, enabling consistent and stable tracking performance.

### 3.2. Experiment

**Figure 8:**
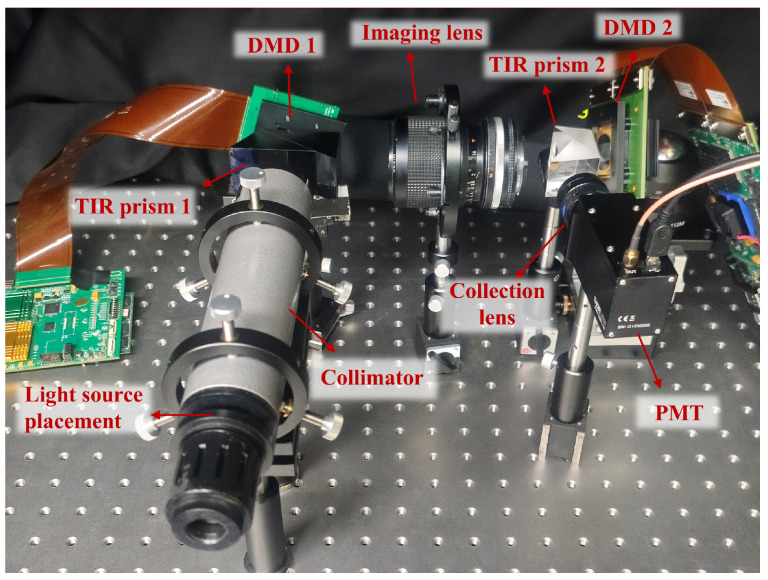
Image-free mult-target tracking system.

We further validated the proposed method through experiments, and the experimental setup is shown in Figure 8. A light source combined with a collimator is used, producing parallel light that is projected onto the micromirrors of DMD1 (DLP700, Texas Instruments, headquartered in Dallas, TX, USA). DMD1 has a resolution of 1024 × 768 and a maximum flipping frequency of 22.2 kHz. Thee rapid flipping of micromirrors is used to simulate the light field of multiple moving objects. The optical information of the objects is focused on DMD2 (DLP9500, Texas Instruments, headquartered in Dallas, TX, USA) through the imaging lenses. DMD2 has a resolution of 1920 × 1080 and a maximum flipping frequency of 17.857 kHz, which is used to modulate the illumination light field. The frame loading frequency of DMD1 is set to match the measurement cycle of DMD2. The modulated light is directed by a total internal reflection prism, which is then collected and detected by a photomultiplier tube (PMT1001/M, Thorlabs, headquartered in Newton, NJ, USA). The detected light information is transmitted to the computer (Intel(R) Core(TM) i5-10210U CPU) through a data acquisition system (USB3133A, ART Technology, headquartered in Beijing, China). The reflected light intensity is recorded synchronously using data acquisition software.

**Figure 9:**
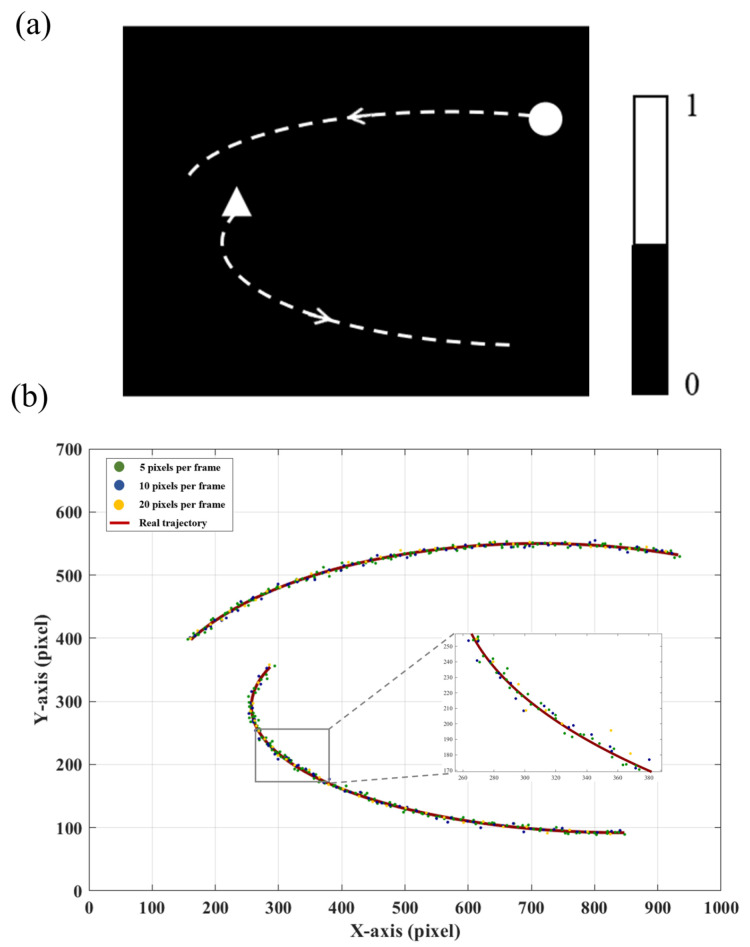
Tracking multiple targets of different speeds. (**a**) Target trajectories. (**b**) Estimation results of targets with different speeds. The two trajectories represent two different moving objects. The green, blue, and yellow points represent the estimated trajectories of the objects moving at speeds of 5, 10, and 20 pixels per frame, respectively, while the solid red line represents the real trajectory.

To test the accuracy of the proposed system in tracking objects with different motion states, we designed tracking experiments with objects moving at different speeds. The objects were simulated using DMD1. The frequency of target image loading was kept consistent in DMD1, and the displacement between two consecutive frames was defined as the object’s speed, measured in pixels per frame. We conducted the tracking on Object 1 (triangle) and Object 2 (circle), and each object occupied an area of approximately 50 × 50 pixels on DMD1. Their motion trajectories are shown in Figure 9a. DMD2 was used for modulation and calibrated with DMD1 for position alignment.

We conducted three tracking experiments. The speeds of the two objects were set at 5 pixels per frame, 10 pixels per frame, and 20 pixels per frame. For each speed scenario, we verified that selecting an appropriate initial smoothing coefficient significantly improves tracking accuracy. As the object speed increased, the initial value of the smoothing coefficient was set to 0.4, 0.5, and 0.7, respectively. A larger smoothing coefficient allows the system to respond more quickly to current measurements, which is beneficial in high-speed scenarios. Meanwhile, the gain factor was kept small to ensure gradual adjustment of the smoothing coefficient, thereby maintaining tracking stability and avoiding abrupt fluctuations due to noise. The real trajectories of the targets and the tracking results at different speeds are shown in Figure 9b. To assess the tracking accuracy of the proposed method, we adopted the normalized root mean square error (NRMSE) as our performance metric. Compared to the root mean square error (RMSE) and mean absolute error (MAE), NRMSE stands out due to its normalization, which is particularly advantageous in high-resolution settings (1920 × 1080 pixels). Moreover, by reflecting errors in proportion to the target’s motion range, NRMSE offers a more balanced evaluation of tracking accuracy. This metric is widely recognized in the tracking literature for its robust interpretability [29], defined as follows:

(20) $$\mathrm{NRMSE}=\frac{\sqrt{\frac{1}{M}\sum_{j=1}^{M}\left({\left(x_{t}\left(j\right)-x_{e}\left(j\right)\right)}^{2}+{\left(y_{t}\left(j\right)-y_{e}\left(j\right)\right)}^{2}\right)}}{\sqrt{\frac{1}{M}\sum_{j=1}^{M}\left(x_{t}{\left(j\right)}^{2}+y_{t}{\left(j\right)}^{2}\right)}}$$

where $\left(x_{t},y_{t}\right)$ represents the true coordinates of the object’s movement, and $\left(x_{e},y_{e}\right)$ represents the estimated coordinates. *M* is the total number of frames. The denominator in the equation normalizes the magnitude of the target’s position, ensuring that the error calculation is unaffected by the target’s location. We calculated the NRMSE at different speeds, as shown in the Table 3. It can be observed that the proposed system achieves stable tracking for multi-target motion scenarios at various speeds.

**Figure 10:**
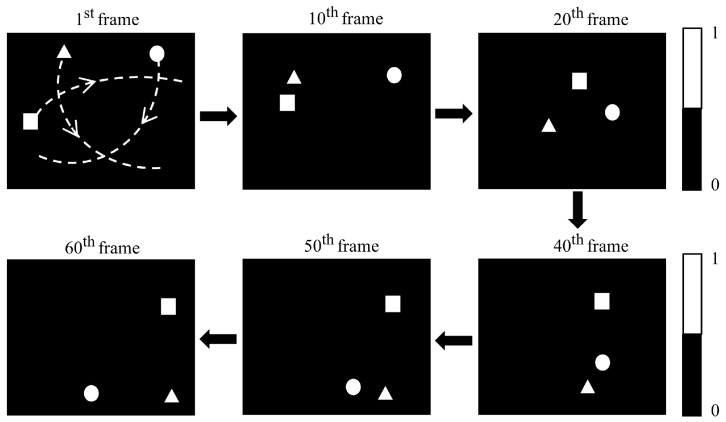
Target trajectories and motion process.

**Figure 11:**
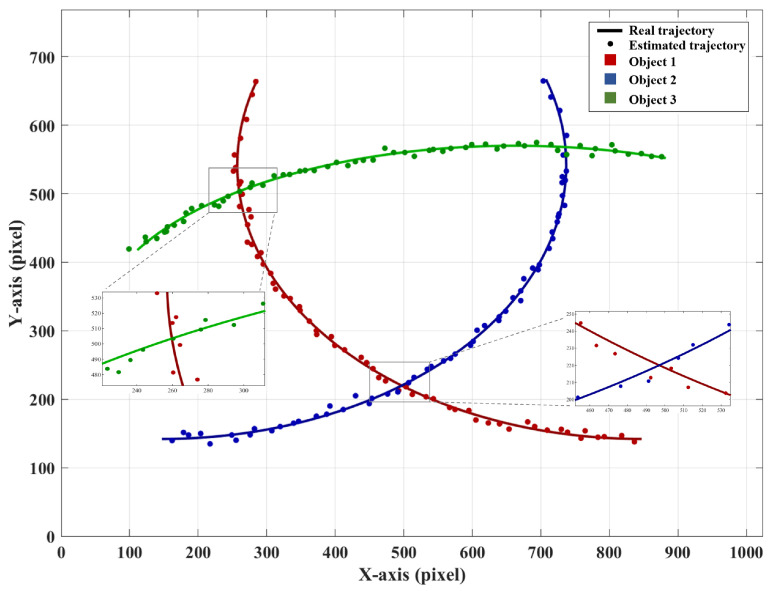
Estimated trajectories in the experiment.

We also experimented with more complex multi-target scenarios. The experiment increased the tracking target to three objects, and their trajectories are shown in Figure 10. Object 1 (triangle) and Object 2 (circle) will move closer to each other and eventually meet, while Object 1 and Object 3 (square) will move closer later and then meet. We selected 60 frames of measurement data, and the estimated trajectory is shown in Figure 11. In the figure, the red line represents the motion trajectory of Object 1, the blue line represents the motion trajectory of Object 2, and the green line represents the motion trajectory of Object 3.

**Figure 12:**
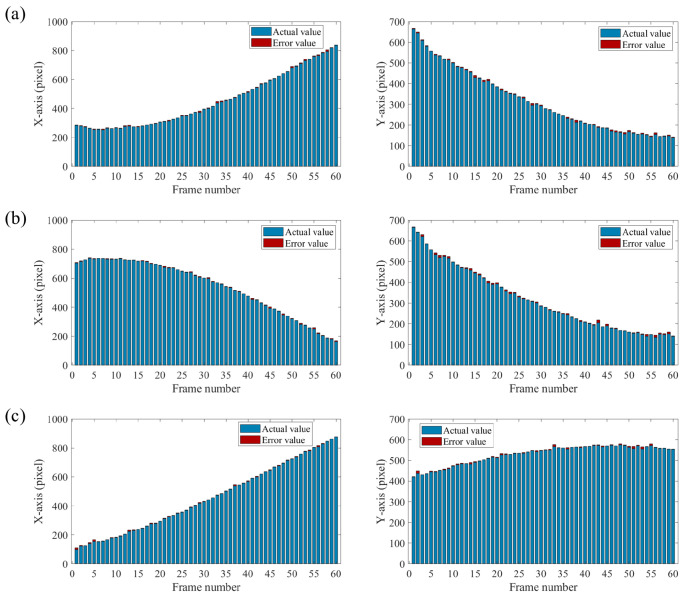
Tracking error of three objects on the x-axis and y-axis. The blue bar represents the actual value, and the red bar represents the error relative to the actual value. (**a**) Object 1. (**b**) Object 2. (**c**) Object 3.

The solid lines represent the real trajectories, while the colored data points correspond to the estimated values. As shown in Figure 11, the discrete trajectory formed by the estimated values matches the real trajectory well. We define the error as the absolute difference between the estimated coordinates and the real coordinates for each data point. The error charts for the *x*-axis and the *y*-axis are shown in Figure 12. It can be seen that the error does not change significantly with the variation in the coordinates. Object 1 and Object 3 meet each other in frame 12, followed by Object 1 and Object 2 meeting each other in frame 38. However, no significant errors are observed during these interactions. During the tracking of three moving objects, only nine masks were needed to obtain the current centroid of each target. Compared with previously reported single-pixel multi-target tracking methods, the proposed approach requires significantly fewer masks, offering a clear advantage in speed. In addition, we provide a more detailed evaluation of tracking accuracy. The NRMSE values for the three objects are calculated as 0.00850, 0.00839, and 0.00785. It can be concluded that the proposed method demonstrates strong robustness in multi-target tracking.

## 4. Discussion

During window-based tracking, keeping the window center close to the target centroid enhances both localization accuracy and tracking stability. To precisely adjust the window position, we introduced a weighting parameter α to balance the influence of the measured centroid $P_{obj}\left(t_{i}\right)$ and the predicted centroid $P_{obj}^{\prime }\left(t_{i+1}\right)$. Experiments show that when target motion is smooth, assigning a higher weight to the prediction yields better accuracy. In contrast, during abrupt motion, relying more on measurements proves more effective. The adaptive adjustment of α is thus critical for robust tracking.

When targets are in close proximity, we jointly estimate target positions and incorporate EWMA smoothing to enhance localization accuracy, enabling continuous tracking. However, the proposed method experiences a sharp increase in tracking error when occlusion occurs between targets, due to the loss of reliable centroid information. Addressing this limitation will be a key focus of future work, with efforts directed toward developing occlusion-resilient models for more robust multi-target tracking performance. Furthermore, since the proposed method relies on real-time control of the DMD for dynamic mask generation, this control latency was not accounted for in the current experimental results. Future research will focus on practical application and propose corresponding optimization strategies to address this limitation.

## 5. Conclusions

In this paper, we presented an adaptive EWMA-based multi-target tracking system using a single-pixel detector, achieving significant reductions in mask utilization while enhancing tracking update rates. By leveraging geometric moment theory, the proposed method resolves target centroids with only 3N measurements for *N* objects, outperforming conventional localization approaches in both computational efficiency and dynamic adaptability. Furthermore, the integration of EWMA enables continuous weight parameter updates through positive feedback, ensuring robust adaptation to diverse motion patterns and environmental perturbations. Numerical simulations reveal tracking accuracies of 0.7 pixels under complex motion states and 1.1 pixels in near-interference scenarios, validating the method’s resilience to target proximity. Experimental validation, conducted using a DMD operating at 17.857 kHz, achieves stable tracking of three moving targets with an NRMSE of 0.00785 under 1920 × 1080 pixel resolution. These results underscore the capability of the proposed system, which maintains high-speed detection and high-precision tracking in scenarios involving multiple targets with complex and time-varying trajectories.

## Figures and Tables

**Table 1:** Initial localization results using Radon projection.

Objects	Object Centroid Value (pixel)	Window Function Center Value (pixel)
Moon	(200, 212)	(200.50, 258.00)
Plane	(794, 366)	(783.25, 358.50)
Car 1	(212, 925)	(209.25, 918.50)
Car 2	(730, 903)	(742.50, 908.00)

**Table 2:** Estimation results of two methods.

Objects	WGM Method Without EWMA RMSE (pixel)	EWMA-Based WGM Method RMSE (pixel)
$N_{1}$	1.21	0.73
$N_{2}$	16.29	1.17
$N_{3}$	1.47	1.16
$N_{4}$	N.A.	1.57

**Table 3:** Estimation results of objects at different motion speeds.

Objects	NRMSE at Motion Speed of *S* Pixels per Frame		
	*S* = 5	*S* = 10	*S* = 20
Object 1	0.00618	0.00694	0.00722
Object 2	0.00783	0.00814	0.00903

## Data Availability

The data presented in this study are available from the corresponding author upon reasonable request due to confidentiality requirements associated with the project.

## Abbreviations

The following abbreviations are used in this manuscript:

EWMA	Exponentially weighted moving average
WGM	Window-based geometric moment
NRMSE	Normalized root mean square error
DMD	Digital micromirror device
PMT	Photomultiplier tube
TIR	Total internal reflection
